# Association of Microbiome with Oral Squamous Cell Carcinoma: A Systematic Review of the Metagenomic Studies

**DOI:** 10.3390/ijerph18147224

**Published:** 2021-07-06

**Authors:** Lee Su Mun, See Wye Lum, Genevieve Kong Yuiin Sze, Cheong Hock Yoong, Kwek Ching Yung, Liong Kah Lok, Divya Gopinath

**Affiliations:** 1 School of Dentistry, International Medical University, Kuala Lumpur 57000, Malaysia; lee.sumun@student.imu.edu.my (L.S.M.); see.wyelum@student.imu.edu.my (S.W.L.); genevieve.kongyuiin@student.imu.edu.my (G.K.Y.S.); cheong.hockyoong@student.imu.edu.my (C.H.Y.); Kwek.chingyung@student.imu.edu.my (K.C.Y.); Liong.kahlok@student.imu.edu.my (L.K.L.); 2 Oral Diagnosis and Surgical Sciences Division, School of Dentistry, International Medical University, Kuala Lumpur 57000, Malaysia

**Keywords:** oral cancer, microbiome, bacteriome, oral microbiota, metagenomics, microbiota, systematic review

## Abstract

The past decade has witnessed a surge in epidemiological studies that have explored the relationship between the oral microbiome and oral cancer. Owing to the diversity of the published data, a comprehensive systematic overview of the currently available evidence is critical. This review summarises the current evidence on the metagenomic studies on the oral microbiome in oral cancer. A systematic search was conducted in Medline and Embase databases to identify original studies examining the differences in the oral microbiome of oral cancer cases and controls. A total of twenty-six studies were identified that reported differences in microbial abundance between oral squamous cell carcinoma (OSCC) and controls. Although almost all the studies identified microbial dysbiosis to be associated with oral cancer, the detailed qualitative analysis did not reveal the presence/abundance of any individual bacteria or a consortium to be consistently enriched in OSCC samples across the studies. Interestingly, few studies reported a surge of periodontopathogenic taxa, especially *Fusobacteria*, whereas others demonstrated a depletion of commensal taxa *Streptococci*. Considerable heterogeneity could be identified in the parameters used for designing the studies as well as reporting the microbial data. If microbiome data needs to be translated in the future, to complement the clinical parameters for diagnosis and prognosis of oral cancer, further studies with the integration of clinical variables, adequate statistical power, reproducible methods, and models are required.

## 1. Introduction

Oral squamous cell carcinoma (OSCC), commonly referred to as oral cancer, is the eighth-most prevalent cancer universally and has a 5-year survival rate of less than 50% [1]. In the United States alone, a total of 53,260 new cases and 10,750 deaths were projected for oral and oropharyngeal cancer in 2020 [2]. The global burden of oral and oropharyngeal cancer on the healthcare system is evident, hence, a clear systematic method of identifying oral cancer at the earliest possible stage is essential, which can ensure prompt treatment administration and higher cure rates. Principal risk factors for oral and oropharyngeal cancers include tobacco use and alcohol consumption [3]. Comparatively, other factors like genetics, oral health, low socioeconomic status, and human papillomavirus (only for oropharyngeal cancer) play a more minor role [4]. A proportion of oral cancers, especially in Asia, arise in the mucosa bearing long-standing pre-existing changes, visible as distinct clinical lesions, which are now collectively termed as “oral potentially malignant disorders (OPMD) [5,6]. 

Recently, multiple research studies have suggested that shifts in microbiota may disrupt the balance between microorganisms and humans, which, when coupled with risk factors, can lead to oncogenesis [7]. It is also hypothesized that bacteria may have a role in carcinogenesis by promoting chronic inflammation, preventing apoptosis, and generating oncogenic substances [8]. Furthermore, several cancers have been directly linked to bacterial infections; for instance: *Helicobacter pylori* and gastric carcinoma, *Salmonella Typhi* and gallbladder carcinoma, *Salmonella Enterica* and colon carcinoma, *Chlamydia trachomatis* and carcinoma of the cervix and ovaries [9]. *Fusobacterium nucleatum* (*F. nucleatum*) and *porphyromonas gingivalis* (*P. gingivalis*) are some of the most studied oral bacteria with oncogenic properties in vitro [8].

In 2007, the National Institutes of Health (NIH) started the Human Microbiome Project (HMP) to study the human microbiome, which is defined as the collective genomes of the microbes within the human body. The role of the microbiome as a whole in various diseases, including oral diseases, has been increasingly recognized. Several studies have individually undertaken the task of collecting samples from oral/oropharyngeal cancer sites and profiling them using next-generation sequencing techniques as an attempt to identify the bacterial community associated with cancer. There have been attempts to assess if microbial dysbiosis, defined as any change in the composition of resident commensal communities, can be regarded as a causative factor or sequelae to oral/oropharyngeal cancer. However, the term “dysbiosis” is inconsistently and often vaguely utilized with a broad range of stochastic interpretations [7]. Overall, our current insights into the exact relationships between the oral microbiome and OSCC remains limited, and a meaningful consensus has yet to be reached regarding cancer-associated changes in species abundance and diversity. It is unclear whether we can identify individual microbes or microbial signatures consisting of a group of microorganisms that are consistently depleted or elevated in OSCC across the patients. Thus, the current article aims to compile the updated evidence regarding the bacterial association with OSCC with a systematic review of published epidemiological studies that have investigated an association of the oral microbiome and OSCC.

## 2. Materials and Methods 

### 2.1. Design

A systematic literature search was conducted independently by two authors (S.W.L., L.S.M.) to identify original observational studies examining the differences in the oral microbial community as a whole in oral samples in patients with oral cancer and healthy controls using next-generation sequencing techniques. This systematic review adheres to the PRISMA (Preferred Reporting Items for Systematic Reviews and Meta-Analyses) guidelines.

### 2.2. Data Sources and Search Strategy

The strategies and criteria of inclusion were discussed among the authors beforehand. Published literature was systematically searched without date limitations until October 2020, using specific search terms through two databases, Medline and Embase, to discover articles related to oral cancer and oral microbiome. The search strategy comprised of the following words: “mouth neoplasms”, “oral carcinoma”, “OSCC”, “oral malignancy”, “uncontrol growth”, “bacteria”, “microbiota”, “microbiome”, “metagenomics”, “metagenome”, “sequence analysis”, “DNA”, “RNA”, “ribosomal”, and, “16S”. Boolean operators “AND” as well as “OR” were used for more focused and productive results. The results were again confined to the English language and humans. The detailed search strategy is provided in Supplementary Table S1. Manual searches for references of the included articles were also conducted to avoid the omission of relevant articles. The authors initially screened the articles for eligibility based on the titles and abstracts, and subsequently, the selected articles underwent a full-text review.

### 2.3. Eligibility Criteria

The studies included were original observational studies that met the following inclusion criteria;

Inclusion criteria: Studies that investigated the oral microbiome by profiling the genome of the whole microbial community through the metagenomic sequencing of oral samples from OSCC cases, relative to controls.

Exclusion criteria: Culture-based studies, papers from conferences or congresses, systematic reviews, and case reports were excluded. Articles other than the English language and human subjects were again excluded.

The PICO question for the review was as follows: 

Population: Patients with OSCC

Intervention: Metagenomic sequencing studies which investigated the whole oral microbial community (microbiome).

Control: Healthy control samples without any clinical or histological evidence of OSCC.

Outcome: Microbial diversity and the relative abundance of various oral bacteria.

### 2.4. Study Selection and Data Extraction

Two authors screened the studies and those that did not meet the inclusion criteria were excluded. Any disagreement between authors was resolved through discussion. Data extraction was undertaken by specified authors using a pre-designed data extraction excel sheet. The following parameters were collected from each study:

- study characteristics—author, year, country, study design, sample details;
- outcomes—diversity and richness, relative abundances of various taxa and microbial functions;
- methodology—DNA extraction, amplification, sequencing platforms, and reference.

### 2.5. Risk of Bias Assessment

The adjusted Newcastle–Ottawa Scale was used to assess the risk of bias, as described previously [10,11], as the selected studies were non-randomized case–control studies [10,11] (Supplementary Table S2).

## 3. Results

### 3.1. Study Characteristics 

**Figure 1:**
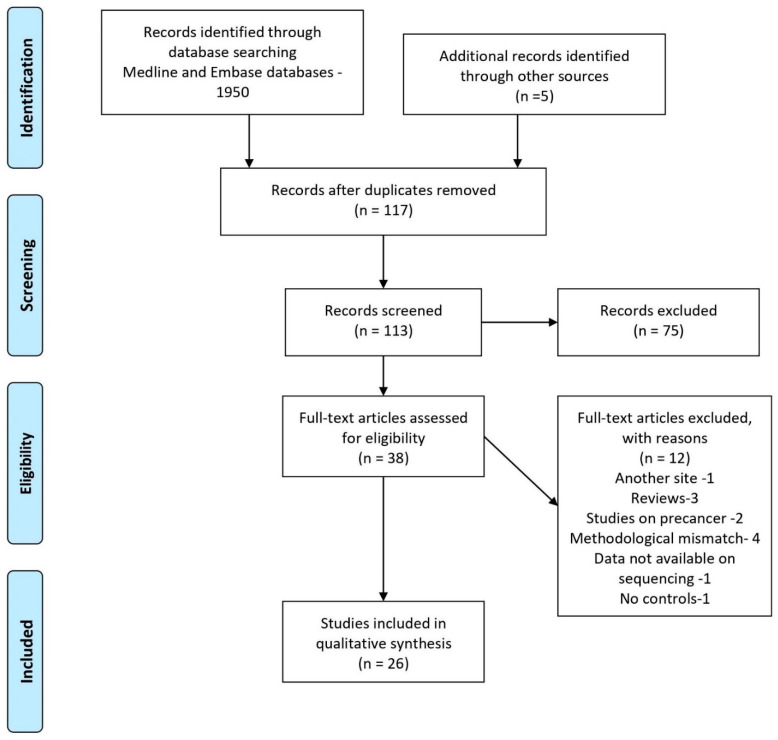
PRISMA flow chart for the selection of studies.

A total of twenty-six articles were included for review; a detailed flow chart of the selection process is provided in Figure 1. The characteristics of the selected studies are described in detail in Table 1. Of the twenty-six studies, ten were from the United States of America [12,13,14,15,16,17,18,19,20,21], four were from China [22,23,24,25], three were from Taiwan [7,26,27], two were from India [28,29], and others were from Yemen [30], Malaysia [31], Australia [32], Japan [33,34], Sri Lanka [35], and New Zealand [36]. The study design for all the studies was cross-sectional, except for one which employed a prospective methodology [19]. The total sample size of the selected studies ranged from 5 to 383, with the number of cases ranging from 3 to 249 and the number of controls ranging from 2 to 242. Collectively, there were 1441 cases and 1368 controls represented in a total of 26 publications. The average age of the subjects ranged from 18 to 81 years. The majority of the studies included both genders as the subjects except two studies which were restricted to male subjects only [12,35]. Other associated factors such as cigarette smoking, betel quid chewing, and alcohol use, HPV, etc., were investigated in fifteen of the selected studies [7,12,20,21,23,24,26,27,28,29,30,31,32,34,35]. The ability to compare or incorporate the results of individual studies was restricted by the extensive differences in various aspects of the studies. 

### 3.2. Sample Collection and Measurement

Different types of samples were used to characterise the oral microbiome found in patients with oral cancer, as described in Table 1, including saliva [12,14,26,27,28,33,34,36], swabs from the oral cavity [13,22,29,31], the tumor tissue [14,16,19,23,24,25,30,35], oral brushings [21], and oral rinses [15,17,20,32]. Samples from normal healthy individuals, as well as the patient’s own mucosa, were utilized as controls in the included studies. Those studies which used samples from normal healthy individuals as controls had varied matching criteria which included site [12,28,30,33,35], age [26,30], and gender [12,30,35]. The microbiome from the tumor tissues was compared with the adjacent non-tumorous tissues from the same patients [16,19,22,24,25], non-tumorous tissues from the contralateral side of the same patients [16,24], with fibroepithelial polyps from healthy controls [35], and healthy tissues from normal healthy individuals [23,30]. Salivary samples utilized included stimulated saliva samples [12,14,26,27,28,33,34,36] and unstimulated saliva samples (29,36) from patients with OSCC compared with those from healthy controls. Few studies utilized oral swabs from oral lesions compared with those from the contralateral normal regions of the oral cavity [13,22] or with the oral swabs from normal healthy individuals [31]. Other types of samples used were oral brushings from buccal mucosa in both cases and controls [21], oral rinses from patients with oral cavity cancer compared with normal healthy individuals [15,17,32], or oral rinses from patients with oral cancer compared with normal healthy individuals [20].

### 3.3. Techniques of DNA Extraction and Sequencing

#### 3.3.1. DNA Extraction

The numerous methods that were employed to extract DNA from the oral samples are listed in Table 2. The different kinds of commercial DNA kits used were: DNA Purification Kit (Epicenter) [12], RNeasy Mini, RNA Isolation Kit [19], DNeasy Blood and Tissue Kit (Qiagen) [13,28,29], DDK DNA Isolation Kit [30], All Prep DNA/RNA FFPE Kit [16], QIAsymphony Virus/Bacteria Midi Kit [17], PowerSoil DNA Isolation Kit, MoBio [18], QIAamp DNA Mini Kit [22], QIAamp DNA Blood Mini Kit (Qiagen) [26], QIAamp DNA Microbiome Kit [7], QIAampFast DNA Stool Mini Kit [23], QIAGEN QIAamp MinElute Virus Spin Kit [27], Commercial Kit (EURx) [31], Maxwell^®^ 16 LEV blood DNA Kit [32], Gentra Puregene Tissue Kit (Qiagen) [35], DNA Purification Kit (Qiagen), and TIANamp Swab DNA Kit [24]. In addition, DNA extraction by the traditional phenol-chloroform method was also utilised by three studies [14,15,36].

#### 3.3.2. DNA Amplification, Sequencing, and Reference Databases

DNA amplification has been carried out by targeting different hypervariable regions of bacterial 16S rRNA genes in these studies. Some study focused only on a single variable region such as V4 [13,17,19,21,26,33] while some focused on multiple regions for instance V1–V3 [30,35], V3–V4 [7,18,20,23,24,25,28,29,34,36], V3–V5 [14,15,27], V4–V5 [12,22], V6-V8 [32], and V6-V9 [31]. After DNA amplification was completed, DNA sequencing was implemented. The majority of these studies carried out sequencing by using the Illumina MiSeq system [7,16,17,21,22,23,24,26,28,29,30,32,33,34,35,36]. The second most common technique used was 454 pyrosequencing [12,13,14,15,18,20]. The Illumina PE250 platform was used by Zhou et al. 2020 [25]. Several different reference databases were utilized for sequencing alignment including GenBank [31], Ribosomal Database Project (RDP) [12,14,15,16,22,24,27], Human Oral Microbiome Database (HOMD) [7,18,30,33,36], Greengenes [13,16,19,20,21,25,28,29,32], SILVA [26,34], NCBI [7,23], BLASTN [35], and Resphera Insight [15].

### 3.4. Microbial Diversity and Abundance

Diversity can be categorized into alpha diversity and beta diversity. Alpha diversity is a local measure that is comparable within samples. In contrast, beta diversity shows differences in the composition of organisms among different individuals. In our review, only twelve out of twenty-six articles reported the diversity between diseased and healthy controls, regardless of healthy humans or healthy samples from cancer patients. Two articles did not report any significant differences in microbial richness and diversity between the cancer groups and control groups [30,31]. Four studies discovered greater richness and diversity in cancerous tissues or samples [21,22,24,34]. On the other hand, higher richness and diversity in controls were reported in another six studies [12,14,19,28,32,35]. Among smokers, it was found that patients with head and neck cancer had lower richness, but higher interindividual microbiome variation compared to healthy controls [21].

### 3.5. Microbial Abundance

#### 3.5.1. Phyla

Most of the studies identified Firmicutes as the most abundant phyla in each subgroup (cancerous and healthy) in comparison with other phyla, including *Proteobacteria, Bacteroidetes*, and *Fusobacteria*. These phyla were also discovered in high proportions in cancer tissues, precancerous tissues, and subgingival plaque of OSCC patients [23]. In comparison to cancer samples, phylum Firmicutes was found to have a lower abundance in oral tissues samples of healthy individuals [19]. Another study reported that phylum *Bacteriodetes* were found more commonly in oral rinse samples of healthy controls in comparison with patients who were diagnosed with oral cavity or oropharyngeal cancers [16]. Another study reported that phylum *Bacteroidetes* was predominant in the OSCC group when compared to the oral leukoplakia group [33].

#### 3.5.2. Classes and Family

Very few studies have described the microbial profile in terms of classes. At the class level, the predominant bacteria in all saliva samples (cancerous, healthy) were *Betaproteobacteria, Bacteroidia, Actinobacteria, Bacilli, Fusobacteriia, TM7-3, Clostridia, and Gammaproteobacteria* [28]. A higher relative abundance of family *Comamonadaceae* was reported in oral cancer cases from the North American cohort [21].

#### 3.5.3. Genera

Numerous studies have reported the presence of bacteria from different genera in various types of samples from diseased patients and disease-free controls. Streptococcus was found to be the most predominant genus across cancer patients and healthy controls in several studies. Few studies stated that genus *Streptococcus* showed the greatest abundance in healthy controls [28,31]. *Fusobacterium* was reported to be abundant in cancer patients by quite a number of studies [7,13,15,20,22,23,24,25,27,29,30,32,34,35]. Genus *Prevotella* was found to be another one of the most abundant genera in cancer patients [27,29,35].

#### 3.5.4. Species

Only a few studies had reported microbiome abundance at the species level. The higher abundance of *Prevotella melaninogenica* and *Veillonella parvula* in cancerous tissues was also reported by Rai et al. 2020 [29]. A study from Taiwan discovered that the saliva of patients diagnosed with OSCC exhibited a predominance of *Prevotella tennerae, F. nucleatum* and *Prevotella intermedia* but a lower abundance of *Streptococcus tigurinus* [27]. In the oral rinses taken from subjects with head and neck cancer, *Lactobacillus spp., Streptococcus mutans, Fusobacterium nucleatum* and *Parvimonas micra* were in significantly high abundance [15]. Chang et al. stated that the composition of bacterial species was similar in cancerous tissues, paracancerous tissues and subgingival plaques [23].

### 3.6. Microbial Association with other Clinical Factors

Guerrero-Preston et al. previously reported that *Veilonella, Megasphaera*, and *Anaerolinaea* were predominant in HPV-positive tumors and could be potential biomarkers for HPV associated oral cavity cancers [14]. Another study reported that oral rinses of HPV positive oral cancer patients were rich in *Lactobacillus gasseri* and *Lactobacillus vaginalis* [15]. A few studies investigated the association between past smoking habits and the cancer microbiome. However, no consistent effects on microbial proportion could be noted. Other clinical and environmental factors, including alcohol consumption and betel nut use, did not show any significant evidence of associations with oral cancer [17]. Takahashi et al. discovered a greater abundance of *Peptostreptococcus* and a reduced proportion of *Haemophilus* in saliva samples of females in comparison with males [34].

### 3.7. Microbial Functions

Seven studies reported the predicted functions of the microbiome with the help of advanced bioinformatics software. These programs help to identify the potential functions of these microbes from the whole genome sequences in the established databases. Three studies revealed a notable increase in lipopolysaccharide synthesis in the microbiome associated with oral cancer [17,30,35], whereas four studies reported alterations in amino acid metabolism [7,22,25,28]. These have been listed in Table 3.

Two studies reported an increase in genes associated with glucose metabolism in the control groups [30,35]. On the contrary, Yang et al. found carbohydrate metabolism to increase with OSCC staging [7]. Sharma et al. reported bacterial metabolic pathways mainly involved in amine and xenobiotic degradation to be more prevalent in cases and sugar degradation pathways in controls [21]. Zhao et al. reported the downregulation of pathways related to membrane transport and upregulation of genes associated with cytoskeletal proteins in oral cancer [22].

## 4. Discussion

Oral cancer has been one of the most pervasive diseases known to the human species, with OSCC representing 90% of the cases. Although the oral cavity harbors an estimated 500 to 700 microorganisms of different species, there is inconclusive evidence on the relationship between microbiota and oral cancer [37]. In this systematic review, our objective was to critically review the studies that investigated the association of the oral microbiome with oral cancer through DNA sequencing of oral samples. Our systematic review is partly attributed to the hypothesis that certain microbial populations may be associated with the pathogenesis of oral cancer and, thus, can be utilized as an indicator for oral malignancy.

Overall results in comparison of diversity and richness between healthy and tumor tissues showed inconsistency. Microbial diversity compared between malignant and healthy tissues within the same sample showed similarity. Conversely, samples isolated from different cases and control samples displayed significant differences. However, the data obtained were not unforeseen. The concept of field cancerization can be a plausible explanation for similarities identified in the resident microbiome adjacent to premalignant or malignant tissues [38]. It is generally deemed that there is a reduction of microbial diversity in cancers, and a more diverse microbiome is associated with health [10]. However, we did not find a similar observation with microbiome studies in oral cancer. The diversity of the oral cavity environment consisting of different complex sub-niches that harbor divergent resident microbiota could be a reasonable explanation [39].

The differences in the abundances of phyla *Fusobacteria, Firmicutes*, and *Bacteroidetes* were predominant in several studies. *Fusobacteria* was targeted in many studies due to its potential role in colorectal cancer occurrence and progression through stimulating cell proliferation, increasing cellular migration and invasion, and inducing inflammation [40]. In addition, stimulated production of IL6 and activation of STAT3 during the incubation of *F. nucleatum* on OSCC cells enhanced proliferation and invasion of the cells [27]. However, the consistent presence of *Fusobacterium* could not be detected among the reviewed studies.

The use of consistent diagnostic criteria for the case definition was lacking among the included studies. Although samples from most of the included studies were microscopically confirmed as OSCC, the diagnostic criteria for OSCC were poorly described. The utilization of international diagnostic classification standards for all clinical and research purposes is recommended for more comparable results. The pooling of samples from the oral cavity with those from the pharynx and larynx can produce significant bias as the differences identified may be due to the microbial variations corresponding to the diverse sites [41]. The sampling strategy is to be considered carefully as different samples may hinder comparison, as the oral microbiome may differ according to the type of samples [10]. The surface samples may depict colonizing microbiome, whereas deeper tissue samples might reflect more significant microbiota that may play a potential role. Salivary samples may be reflective of the total oral environment, whereas direct sampling from tissue samples may be more representative of an endogenous microbiome co-evolving with the host [42]. The microbial communities collected from the mucosal surface by an oral swab may not reflect the tumor-associated microbiome [43,44]. The surface microbial communities may also be influenced by various factors, including salivary pH, redox potential, and caries/periodontal status. Salivary samples could be utilized for exploring biomarkers as predictive models using multiple bacteria. Multiple multi-bacterial predictive models using the fecal microbiome have been reported to distinguish colorectal cancer patients from healthy controls, which has the potential to be validated in a new population [45]. However, only a single study has reported the utility of an oral microbiome panel in discriminating oral and oropharyngeal cancer patients from normal healthy individuals [32].

The reliability of microbiome studies largely depends on the molecular biology techniques utilized downstream. Hypervariable regions of the 16S rRNA gene and sequencing platforms play an important role in influencing the end results of the studies [46]. Most studies sequenced the V3–V4 regions, although some chose V4, V3–V5, V4–V5, and V6–V8 regions. Experimental studies have concluded that the type of 16S rRNA region chosen for amplification can significantly affect the proportions of distinct taxa [10]. Apart from the choice of hypervariable regions of the 16S rRNA, the database and classifiers used will also add to the technical differences in the microbiome data [10]. Although 16S rRNA gene amplicon sequencing is cost-effective, it only provides taxonomic classification up to the genus level [47]. Characterizing the data at the genera level necessarily constrains the biological interpretations of categorized associations, as several species or even strains under the same genera may have a different impact on a particular disease.

Several different oral bacterial species have been shown to promote cell proliferation. *P. gingivalis* has been involved in the downstream signaling pathway of the transcription factor NF-κB and few MAPK family members including MAPK8 and MAPK14 that play an important role in oncogenesis [48]. *F. nucleatum* has also been shown to upregulate the Toll-like receptor (TLR) signaling and activation, of cell cycle regulators STAT3 and cyclin D1, leading to the growth of cancer cells [49]. Apart from cell proliferation, certain oral bacterial species have been shown to indirectly inhibit apoptotic pathways and increase the survival of cells [50,51,52]. In vitro studies have also demonstrated the impact of *P. gingivalis* and *F. nucleatum* on the upregulation of matrix metalloproteinases, including MMP-2, MMP-3, and MMP-9, which degrade the extracellular matrix and the basement membrane enabling cancer cells to invade and translocate to other sites [53]. Hence, metagenomic and meta-transcriptomics approaches to improve the taxonomical, as well as functional resolution, are the way forward.

Analyzing the results of the sequencing studies demonstrate a highly complex diversity in the oral microbiome associated with oral mucosal diseases. There has not been any consensus regarding a single genera or species that could be useful for discriminating between health and oral cancer. Therefore, comparisons of complexes of microorganisms or community-level comparisons are now being included in the analysis. Collectively, microbiome studies have established that the oral microbiome in cancer patients differs from healthy controls. Many studies have demonstrated a shift towards gram-negative bacteria which have been implicated in the pathogenesis of periodontitis, as illustrated in Table 1. The presence of periodontal disease is one of the most important confounding factors which can bring potential bias in microbiome studies on OSCC. Periodontal disease and oral cancers are both diseases of the elderly. There is increasing evidence for periodontal disease to be considered as a putative risk factor for oral cancer [4]. The possible link between these two is inflammation which is considered as the seventh hallmark of cancer [54]. The inflammatory mediators released in response to the periodontopathogenic bacteria, as well as their compositional and metabolic products, are well-known activators of pathogen recognition receptors such as toll-like receptors [55]. The prolonged exposure of mucosa to numerous chemokines and other inflammatory mediators released in chronic periodontitis may promote a favorable environment by establishing DNA damage, thereby contributing to tumorigenesis. Hence, it seems logical that the functional component of the oral microbiome is playing a more inevitable role than the phylogenetic composition. Microbial studies have also shown that an increase in lipopolysaccharide synthesis and altered amino acid metabolism in the microbial community in oral cancer [35]. Nevertheless, it is uncertain whether this shift can be considered as a precursor step or opportunistic colonizing of these organisms from the gingival pockets to a more enriching microenvironment. Thus, elucidating the exact role of the microbiome in the initiation and progression of oral carcinogenesis can be challenging owing to the complex niches in the oral cavity. Temporal profiling of the microbiome of potentially malignant disorders as well as their periodontal parameters longitudinally is a possible way forward to unravel this complex mystery.

A recent meta-analysis of the gut microbiome highlighted the concept of a nonspecific microbial response to be considered in all the future case–control oral microbiome studies [56]. It has been suggested that results from microbiome studies should be viewed with caution, especially for cancer studies, as most of the reported microbiome association could be suggestive of a shared response to a common symptom (ulceration, inflammation) of cancer and health rather than a cancer-specific biological difference [56]. Health-associated nonspecific bacteria are usually ubiquitous and abundant across the population, whereas disease-associated bacteria are abundant when present in disease, but not ubiquitous to the entire population. The most ubiquitous and common commensal colonizers, as well as periodontopathogenic bacteria that respond to or cause local inflammation, can be frequently present in the oral cavity of healthy controls and maybe overrepresented in periodontitis. Hence, attempts should be made to identify subsets exhibiting distinct microbial dysbiosis without such confounders to further decode the microbial–host interactions.

The microbiome may also play a plausible role in the progression of cancer, including the differentiation of the tumor, its local spread and invasiveness, as well as distant metastasis. Periodontal inflammation has been shown to induce epithelial–mesenchymal transition, which is an important element of tumor invasiveness as well as secretion of the angiogenic factors VEGF and angiogenin [57,58]. Therefore, clinical studies should gauge the association of the oral microbiome and other tumor characteristics like a lymphatic and perineural invasion. Moreover, parallel evidence is also required from murine studies in cancer, in which gnotobiotic mice, chemically induced or genetically predisposed to oral carcinogenesis are used to detect whether carcinogenesis can be potentiated by exposing mice to specific bacteria or saliva from oral cancer patients. A recent study using germ-free mice illustrated that the presence of various bacterial taxa enhanced tumorigenesis potential and enhanced the number of tumors in the mice [59]. Moreover, the community-wide metabolic profiles of the microbiome showed that the same metabolic activities were consistently associated with OSCC irrespective of the microbial composition [59].

## 5. Conclusions 

Based on current evidence, we can conclude that there is significant dysbiosis in the phylogenetic composition of the oral microbiome on oral cancer patients. However, there aren’t any particular genera or species of bacteria to be highlighted to have a significant contribution to oral tumorigenesis. It could be hypothesized that a critical element in elucidating the contribution of oral microbiome to oral carcinogenesis would be the collective functions of the microbial community, thus accounting for the absence of a consensus on the microbial profile in OSCC.

Hence, a functional approach through meta-transcriptomics might be the way forward to identify the contributory role of the oral microbiome in oral carcinogenesis and its influences on the behavior of the neoplasm. In addition, host-microbial interactions could also pave the way in enhancing our understanding of the tumor’s microbial community. The oral microbiome has been known to exhibit variations between individuals and within the same individual. Further, we have a limited understanding of the dynamics of the oral microbiome as well. Thus, without baseline data on the oral microbiome of the same individual in health, the translational aspect of cancer microbiome studies might still be inconclusive. Moreover, it is also important to consider the role of phages, archaea, and fungi in oral health and diseases. Functions and potential roles need to be explored as research into those topics are still in the infancy stage and functional evidence is essential to expand the current insights into meaningful conclusions.

Translation of murine studies to humans is also challenging as the microbiome significantly differs between humans and mice. Standardization and repeatability of oral microbiome research is another question that calls for researchers to work on a global level for standardization in oral microbiome research. Therefore, it is fair to presume that oral microbiome research, unlike gut microbiome research, is still far away from translation; more systematic studies with integrated methods are needed to determine the potential mechanisms and role of the oral microbiome in oral cancers and other diseases. Even though we have accumulated evidence on the strong association between microbiome and cancer, we also need to expand the microbiome research into bacterial species and genes to gain insight into the exact role of microorganisms in the causality as well as the progression of a tumor.

If the potential involvement of the oral microbiome in the progression of oral cancer can be completely elucidated, analysis of the microbiome would become a useful indicator of the efficacy of chemotherapy, radiotherapy, and immunotherapy. Collectively, all the observational studies have offered an invaluable understanding of oral microbiome composition in oral cancer patients; however, if we are to translate this for clinical use, we should work on developing our understanding of the utility of oral microbiome manipulation by emphasizing interventional research with clinical impact.

## Figures and Tables

**Table 1:** Summary of characteristics of epidemiological studies on the microbiome and oral cancer.

No.	Author, Year	Sample Type	Age (Mean/Median)	Nature of Control	Study Population Size (Case, Control)	Other Clinical Features Studied	Results: Diversity and Richness	Bacterial Taxa Associated with Tumors and Controls
1	Pushalkar et al. 2011 [12]	Saliva	Age: >50	Healthy controls	Case: 3 Control: 2	Smoking: at least one pack of cigarettes a day. Alcohol: more than five drinks a day	Increase in diversity in the control group	*Streptococcus, Gemella, Rothia, Peptostreptococcus, Lactobacillus, Porphyromonas* in OSCC group. *Prevotella, Neisseria, Leptotrichia, Capnocytophaga, Actinobacillus* and *Oribacterium* in the control group.
2	Schmidt et al. 2014 [13]	Oral swab	Cancer: 62 Control: 31	Contralateral normal regions of the oral cavity	Case: 50 Control: 20	N/A	N/A	Decreased relative abundance of *Streptococcus* and *Rothia* in the tumor group. Increased relative abundance of *Fusobacterium* in the tumor group.
3	Guerrero-Preston et al. 2016 [14]	Tissue and saliva	OSCC: 66 OPSCC: 62 Control: N/A	Healthy controls without smoking and drinking	Case: 19 Control: 25	N/A	Decrease in richness and diversity in cases Significant increase of certain *Lactobacillus* and *Weeksellaceae* in HPV+ samples Significant abundance of *Eikenella, Neisseria*, and *Leptotrichia* in HPV– samples	Significant increase of *Lactobacillus, Strepcoccus*, *Staphylococcus* and *Parvimonas* in HNSCC group. Significant abundance of *Haemophilus, Neisseria*, *Gemellaceae* and *Aggregatibacter* in the control group.
4	Al-Hebshi et al. 2017 [30]	Tissue	Case: 53.6 ± 10.4 Controls: 52.3 ± 8.9	Healthy, gender- and age-matched controls	Case: 20 Control: 20	Smoking: shammah (smokeless tobacco)	Similar species richness and α-diversity in both groups (tissue biopsies)	Significant abundance of *Fusobacterium* in OSCC group. Significant abundance of *Streptococcus* and *Rothia* in the control group.
5	Banerjee et al. 2017 [16]	Tissue (FFPE)	N/A	Adjacent non-tumorous tissues, healthy controls	Cases: 100 Controls: 40	N/A	N/A	Significant abundance of *Proteobacteria Brevundimonas, Actinobacteria Mobiluncus, Frateuria, Caulobacter, Actinomyces and Aeromonas* in OSCC group. Significant abundance of *Actinomyces* in the control group.
6	Bornigen et al. 2017 [17]	Oral rinse	58	Healthy controls	Case: 121 Control: 242	N/A	Increase in diversity in smokers	Significant abundance of *Dialister* in the oral cancer group. Significant decrease of *Actinomycetales* and *Lactobacillales* in the oral cancer group.
7	Guerrero-Preston et al. 2017 [15]	Oral rinse	OSCC: 66 OPSCC: 62 Controls: N/A	Healthy controls	Case: 19 (HNSCC) Control: 25	N/A	*Lactobacillus gasseri/johnsonii, lactobacillus vaginalis* in HPV+ patient	Significant increase of *Lactobacillus spp, Streptococcus mutans, Fusobacterium nucleatum* and *Parvimonas micra* in HNSCC group. Significant decrease of *leptotrichia trevisanii, leptotrichia hofstadii* and *buccalis* in HNSCC group
8	Lee et al. 2017 [26]	Saliva	Cancer Age: 53 ± 10 Control Age: 52 ± 14	Healthy controls	Case: 125 Control: 127	Betel nut chewing history Cigarette smoking history	N/A	Significant increase of *Bacteroides, Escherichia, Cloacibacillus, Gemmiger, Oscillospira* and *Roseburia* in cancer group.
9	Mok et al. 2017 [31]	Oral swab	Age: >20	Healthy controls	Case: 9 Control: 9	Smoking history Alcohol consumption history	Similar species richness between cancer and control group	Significant abundance of *Streptococcus* and *Veillonella* in the control group. Significant abundance of *Neisseria, Gemella* and *Granulicatella* in cancer group.
10	Shin et al. 2017 [19]	Tissue	Age: 59 ± 5.6	Adjacent non-tumorous tissue	Case: 34 Control: 24	N/A	Increase in α-diversity in the control group Decrease in β-diversity in tumor	Significant abundance of *Firmicutes* and *Actinobacteria* in the control group. Significant abundance of *Fusobacteria* in primary HNSCC group.
11	Zhao et al. 2017 [22]	Oral swabbing	Median age: 62	Adjacent non-tumorous tissue	Case: 80 Control: 80	N/A	Increase in diversity in cancer group	Significant increase of *Spirochaetes, Fusobacteria, Bacteroidetes, Mycoplasma, Treponema, Campylobacter, Eikenella, Centipeda, Lachnospiraceae_G_7, Alloprevotella, Fusobacterium, Selenomonas, Dialister*, *Peptostreptococcus, Filifactor, Peptococcus, Catonella*, *Parvimonas, Capnocytophaga* and *Peptostreptococcaceae_XI_G_7* in the cancer group. Significant increase of *Firmicutes, Actinobacteria, Megasphaera, Stomatobaculum, Granulicatella, Lautropia, Veillonella, Streptococcus, Scardovia, Rothia*, and *Actinomyces* in the control group.
12	Hayes et al. 2018 [18]	Oral rinse	Case: 60–70 Control: 60–70	Healthy controls	Case: 129 Control: 254	N/A	N/A	Significant increase of *Actinobacteria* in HNSCC group. Significant decrease of *Parvimonas micra* and *Neisseria sicca* in oral cancer group. Significant decrease of Genus *Corynebacterium* up to order *Corynebacteriales*, genus *Kingella* up to phylum *Proteobacteria, Prevotella nanceiensis, Capnocytophaga leadbetteri* and *Selenomonas sputigena* in HNSCC group. Significant increase of *Actinomyces (oral taxon_170*) in the oral cancer group.
13	Hsiao et al. 2018 [27]	Saliva	Cases: ≥20 Control: ≥20	Healthy controls	Case: 138 Control: 151	Betel nut chewing history Cigarette smoking history Alcohol consumption history Oral hygiene status	Significant increase of *Prevotella intermedia* in alcohol consumers and betel nut chewers Significant increase of *F.nucleatum* in smokers Significant increase of *Prevotella tannerae* and *F.nucleatum* in poor dental care group	Significant increase of *Prevotella tannerae, F. nucleatum* and *Prevotella intermedia* in the cancer group.
14	Lim et al. 2018 [32]	Oral rinse	Case: 65 Control: 20–60	Healthy controls	Case: 63 Control: 20	Smoking history Alcohol consumption history HPV status	Decrease in diversity in the cancer group	Significant increase of *Oribacterium* in OCC and OPC group. Significant decrease of *Rothia, Haemophilus, Corynebacterium, Paludibacter, Porphyromonas,* and *Capnocytophaga* in OCC and OPC group. Significant increase of *Actinomyces, Parvimonas, Selenomonas,* and *Prevotella* in OCC group. Significant increase of *Haemophilus* and *Gemella* in HPV+ group.
15	Perera et al. 2018 [35]	Tissue	Case: Age: 61.00 ± 9.5 Controls: Age: 50.58 ± 13.5	Healthy controls	Case: 25 Control: 27	Betel nut chewing history Smoking history Alcohol consumption history Oral hygiene and periodontal status Missing teeth	Decrease in diversity in cancer group	Significant increase of *Capnocytophaga, Pseudomonas, Atopobium, Campylobacter concisus, Prevotella salivae, Prevotella loeschii, Fusobacterium oral taxon 204, F. nucleatum subsp. polymorphum, Streptococcus dysgalactiae, Citrobacter koseri*, and *Pseudomonas aeruginosa* in OSCC group. Significant increase of *Lautropia, Staphylococcus, Propionibacterium, Sphingomonas, Streptococcus parasanguinis, Streptococcus mitis, Streptococcus sp oral taxon 070, Streptococcus sp oral taxon 423, Streptococcus sp oral taxon 431, Streptococcus agalactiae, Rothia dentocariosa, Rothia mucilaginosa, Lautropia mirabilis, Leptotrichia oral taxon 225*, and *Staphylococcus epidermidis* in control group.
16	Vesty et al. 2018 [36]	Saliva	Case: 49 to 81 Control: 20 to 35	Healthy controls (non-smokers)	Case: 23 Control: 7	Fungal communities concentrations of inflammatory cytokines	Increase in fungal diversity in dentally compromised group. IL-1 beta and *Lachnoanaerobaculum* as well as *Actinomyces* and IL-8 had negative correlations	Significant abundance of *Treponema* in cases. Significant abundance of *Actinomyces* and *Fusobacterium* in controls
17	Yang C. et al. 2018 [7]	Oral rinse	Case Age: 50–60 Control: Age: 35–35	Healthy controls	Case: 197 Control: 51	TNM stage Betel nut chewing history Alcohol consumption history	Significant increase of *F. alocis* in smokers	Significant abundance of *Fusobacterium periodonticum, Parvimonas micra, Streptococcus constellatus, Haemophilus influenza* and *Filifactor alocis* in OSCC (Stage 4) group. Significant abundance of *Haemophilus parainfluenzae, Porphyromonas pasteri, Veillonella parvula* and *Actinomyces odontolyticus* in control group.
18	Chang et al. 2019 [23]	Tissue (FFPE)	Case: 57.4 ± 10.4 Control: 55.4 ± 10.2	Healthy controls	Case: 61 Control: 30	Smoking history	Significant increase of *P. gingivalis* in clinical stage III-IV, low degree of tissue differentiation and lymph node metastasis group.	Significant increase *of F. nucleatum* and *P. gingivalis* in cancer group.
19	Ganly et al. 2019 [20]	Oral rinse	Mean age: 21	Patients with benign or malignant thyroid nodules	Cases: 26 Controls: 12	Smoking history Alcohol consumption history	N/A	Significant abundance of *Alloprevotella, Fusobacterium* and *Prevotella* in OSCC group. Significant abundance of *Streptococcus* in control group.
20	Hashimoto et al. 2019 [33]	Saliva	Case: 51 Control: 31	Healthy controls	Case: 12 Control: 4	N/A	N/A	*Porphyromonas gingivalis* in OSCC group. Significant abundance of *Streptococcus anginosus* in OSCC group.
21	Takahashi et al. 2019 [34]	Saliva	Case: 63.7 Control: 65.1	Healthy controls (40 years of age)	Case: 60 Control: 80	Age Sex Smoking history Alcohol consumption history Denture usage	Increase in diversity in OSCC group Decrease in diversity in OSCC Abundance of *Peptostreptococcus* in females Abundance of *Haemophilus* in males and alcohol consumers	Significant abundance of *Peptostreptococcus, Fusobacterium, Alloprevotella, Capnocytophaga* in OSCC group. Significant abundance of *Rothia* and *Haemophilus* in the control group.
22	Panda et al. 2020 [28]	Saliva	Case: 48–58 Control: 40 to 60	Healthy controls	Case: 15 Control: 10	Betel nut chewing history Smoking (smokeless tobacco) history	Increase in diversity in the control group	Significant abundance of *Rothia mucilaginosa, Aggregatibacter segnis, Veillonella dispar, Prevotella nanceiensis, Rothia aeria, Capnocytophage ochracea, Neissseria bacilliformis, Prevotella nigrescens* and *Selenomonas noxia* in the control group. Significant abundance of *Haemophilus parainfluenzae, Haemophilus influenzae* and *Prevotella copri* in the cancer group. *Streptococcus anginosus* was found only in oropharyngeal cancer tissues.
23	Sharma et al. 2020 [21]	Oral brushings	Cases: 58 Controls: 48	Healthy controls	Case: 27 Control: 24	Smoking	Increase in richness in OSCC group	Higher relative abundance of *Stenotrophomonas ruminococcus* and family *Comamonadaceae* in cases *Tannerella*, *Capnocytophaga, Selenomonas, Veillonella*, and *Kingella*, were higher in controls
24	Zhang et al. 2020 [24]	Tissue	Median age: 61	Adjacent non-tumorous tissue	Case: 50 Control: 50	Betel nut chewing history Smoking history Alcohol consumption history	Increase in diversity in OSCC group	Significant abundance of *F.nucleatum, Prevotella intermedia, Aggregatibacter segnis, Campylobacter rectus, Capnocytophaga leadbetteri, Gemella morbillorum, Peptostreptococcus stomatis, Peptococcus sp.* and *Porphyromonas catoniae* in OSCC group. Significant abundance of *Corynebacterium matruchotii, Granulicatella elegans, Granulicatella adicens* and *Streptococcus oralis* in control group.
25	Zhou et al. 2020 [25]	Tissue	61.1 ± 12.4	Adjacent paracancerous tissue 2 cm around edge of tumour	Case: 24 Control: 24	N/A	N/A	Significant increase of *Fusobacterium, Parvimonas, Peptostreptococcus* and *Streptococcus* in cancer group. Significant decrease of *Arthrobacter, Brevundimonas, Microbacterium, Mucispirillum, Paenibacillus and Streptophyta* in cancer group.
26	Rai et al. 2020 [29]	Saliva	Case: 55.32 Control: 50.38	Healthy controls	Case: 11 Control: 10	Betel nut chewing history Tobacco chewing history Tobacco smoking history Alcohol consumption history Family history of cancer	N/A	Significant increase of *Prevotella melaninogenica, Streptococcus anginosus, Veillonella parvula, Prevotella pallens, Porphyromonas endodontalis, Prevotella nanceiensis, Dialister sp., Campylobacter ureolyticus, Fusobacterium sp., Prevotella nigrescens, Neisseria bacilliformis*, and *Peptostreptococcus anaerobius* in OSCC group. Significant increase of *Neisseria subflava, Veillonella dispar, Rothia dentocariosa*, and *Rothia. Mucilaginosa* in control group. Rare species of *Ruminococcus gnavus, Lactobacillus plantarum, Bacteroides ovatus, Parabacteroides distasonis, Filifactor sp.* and *Dorea sp*. found in saliva of OSCC group.

N/A—not available in the article, HNSCC—head and neck squamous cell carcinoma, OSCC—oral squamous cell carcinoma.

**Table 2:** Summary of techniques of DNA extraction, amplification, and sequencing, and reference databases.

No.	Author, Year	Sample	Method of DNA Extraction	DNA Amplification	Sequencing	Reference Databases
1	Pushalkar et al. 2011 [12]	Saliva	DNA Purification Kit (MasterPure)	V4–V5 region.	454 parallel DNA sequencing	RDP II
2	Schmidt et al. 2014 [13]	Oral swab	DNeasy Blood and Tissue Kit (Qiagen)	V4 region	454 pyrosequencing	Greengenes
3	Guerrero-Preston et al. 2016 [14]	Tissue and saliva	Phenol-chloroform method	V3–V5 region	Roche/454 GS pyrosequencing	RDP
4	Al-Hebshi et al. 2017 [30]	Tissue	DDK DNA Isolation kit	V1–V3 region	Illumina MiSeq	HOMD
5	Banerjee et al. 2017 [16]	Tissue	All Prep DNA/RNA FFPE Kit	NA	Illumina MiSeq	RDP
6	Bornigen et al. 2017 [17]	Oral rinse	QIAsymphony virus/Bacteria Midi Kit	V4 variable region	Illumina MiSeq	Greengenes
7	Guerrero-Preston et al. 2017 [15]	Tissue and saliva	Phenol-chloroform method	V3–V5 region	Roche/454 GS pyrosequencing	RDP and Resphera Insight
8	Lee et al. 2017 [26]	Saliva	QIAamp DNA Blood Mini Kit	V4 region	Illumina MiSeq	SILVA
9	Mok et al. 2017 [31]	Oral swab	EURx commercial kit with modifications	V6–V9 region	NA	GenBank
10	Shin et al. 2017 [19]	Tissue	RNeasy Mini, RNA Isolation Kit (Qiagen)	V4 variable region	Ion Torrent Personal Genome Machine (PGM)	Greengenes
11	Zhao et al. 2017 [22]	Swab	QIAmp DNA Mini Kit	V4–V5 region	Illumina MiSeq	RDP
12	Hayes et al. 2018 [18]	Oral rinse	PowerSoil DNA Isolation Kit (MO BIO)	V3–V4 regions	454 FLX Titanium pyrosequencing system (Roche)	HOMD
13	Hsiao et al. 2018 [27]	Saliva	QIAamp MinElute Virus Spin Kit	V3–V5 regions	Illumina MiSeq	RDP
14	Lim et al. 2018 [32]	Oral rinse	Maxwell^®^ 16 LEV Blood DNA kit	V6–V8 region	Illumina MiSeq	Greengenes
15	Perera et al. 2018 [35]	Tissue	Gentra Puregene Tissue kit (Qiagen)	V1–V3 region	Illumina MiSeq	Species-level taxonomy assignment algorithm (BLASTN)
16	Vesty et al. 2018 [36]	Saliva	Phenol-chloroform based DNA extraction	V3–V4 region	Illumina MiSeq	Greengenes
17	Yang et al. 2018 [7]	Oral rinse	QIAamp DNA Microbiome Kit	V3–V4 region	Illumina MiSeq	Greengenes
18	Chang et al. 2019 [23]	Tissue	QIAampFast DNA Stool Mini Kit	V3-V4 region	Illumina MiSeq	NCBI
19	Ganly et al. 2019 [20]	Oral rinse	Modified QIAGEN DNA Extraction Method	V3 and V4 regions	454 FLX platform	HOMD
20	Hashimoto et al. 2019 [33]	Saliva	NA	V4 region	Illumina MiSeq	Greengenes
21	Takahashi et al. 2019 [34]	Saliva	Gene Prep Star PI-80X device	V3–V4 region	Illumina MiSeq	SILVA 128
22	Panda et al. 2020 [28]	Saliva	Qiagen DNeasy Blood and Tissue Kit	V3–V4 region	Illumina MiSeq	HOMD
23	Sharma et al. 2020 [21]	Oral brushings	DNA Purification Kit (Qiagen).	V4—region	Illumina MiSeq	Greengenes (v 13.8)
24	Zhang et al. 2020 [24]	Tissue	TIANamp Swab DNA Kit	V3–V4 region	Illumina MiSeq	RDP
25	Zhou et al. 2020 [25]	Tissue	NA	V3–V4 region	Illumina PE250	Greengenes (v13.5)
26	Rai et al. 2020 [29]	Saliva	Qiagen DNeasy Blood and Tissue Kit	V3–V4 region	Illumina MiSeq	Greengenes (v 13.8)

RDP—Ribosomal Database Project, HOMD—Human Oral microbiome Database, NCBI—National Center for Biotechnology Information, NA—not available, SILVA—Silva ribosomal RNA Gene Database Project.

**Table 3:** Summary of highlighted Microbial functions.

No.	Author, Year	Sample	Microbial Functions Associated with Tumors and Controls
1	Zhao et al. 2017 [22]	Swabs	Translation, metabolism of cofactors and vitamins, metabolism of terpenoids and polyketides, replication and repair in cases
2	Al-Hebshi et al. 2017 [30]	Tissue	Bacterial mobility, flagellar assembly, bacterial chemotaxis, and LPS biosynthesis in cases DNA repair, glycolysis/gluconeogenesis, and biosynthesis of amino acids in controls
3	Perera et al. 2018 [35]	Tissue	Lipopolysaccharide biosynthesis, peptidases, carbon fixation in photosynthetic organisms in cases Base excision repair, glycolysis/gluconeogenesis, and biosynthesis of amino acids in controls
4	Yang et al. 2018 [7]	Oral rinse	Cytoskeleton proteins, methane metabolism, carbon fixation in photosynthetic organisms, restriction enzymes in cases. Amino acid synthesis and metabolism in controls
5	Zhang et al. 2020 [24]	Tissue	Proinflammatory bacterial component, such as lipopolysaccharide biosynthesis; metabolism of cofactors and vitamins, such as porphyrin and chlorophyll metabolism in cancer cases.
6	Zhou et al. 2020 [25]	Tissue	Methane metabolism, glucose-related metabolisms, such as phosphotransferase system (PTS) and glycolysis, were significantly enriched in cancer cases.
7	Sharma et al. 2020 [21]	Oral brushings	Xenobiotic and amine degradation in cases and sugar degradation pathways in controls

## Data Availability

Not applicable.

## Supplementary Materials

The following are available online at https://www.mdpi.com/article/10.3390/ijerph18147224/s1, Table S1: search results, Table S2: New castle ottawa risk of bias (adjusted).
