# Supplementary material for: Association of Microbiome with Oral Squamous Cell Carcinoma: A Systematic Review of the Metagenomic Studies
Source: Int J Environ Res Public Health. 2021 Jul 6;18(14):7224. doi: 10.3390/ijerph18147224 (PMC8306663; doi:10.3390/ijerph18147224)
Supplement: Supplementary file 1 [file ijerph-18-07224-s001.zip › ijerph-1248618-supplementary.pdf]

Supplementary table S1: Search Results

No	Search Terms	Search Results	
		MEDLINE	Embase
1	exp Mouth Neoplasms/	67956	104768
2	(oral adj2 carcinoma$).ti,ab.	3233	3771
3	(oral adj2 malignanc$).ti,ab.	1	932
4	(uncontrol$ adj2 growth$).ti,ab. and exp mouth/	9	7
5	(uncontrol$ adj2 growth$, mouth).ti,ab.	0	0
6	(uncontrol$ adj2 growth$, mouth).tw.	0	0
7	exp Sequence Analysis, DNA/	225492	199648
8	exp RNA, Ribosomal, 16S/	48922	60121
9	exp Metagenomics/	5533	12588
10	exp METAGENOME/	6033	4906
11	metagen$.tw.	12491	14542
12	(16S or 16 S or 16-S).ab.	65802	74695
13	microbiota/	14981	14092
14	exp BACTERIA/	1342430	1446209
15	(microbio$ or microb$ or bacteri$).ti,ab.	990726	1167773
16	1 or 2 or 3 or 4 or 5 or 6	68869	105842
17	7 or 8 or 9 or 10 or 11 or 12	289992	288888
18	13 or 14 or 15	1815815	1993614
19	16 and 17 and 18	39	109
20	Limit 19 to (english language and humans)	37	101

Supplementary table S2: New castle ottawa risk of bias (adjusted)

No.	Author, year	Selection (Definition and selection of cases and controls ;max = 4 *)	Comparability (cases and controls ;max = 2 *)	Outcome (outcomes measured as relative abundances of bacteria, Same method for cases and controls, statistical analysis ;max = 4 *)	Total (10)
**1.**	Pushalkar et al., 2011	**	*	*	4
**2.**	Schmidt et al., 2014	***	**	**	7
**3.**	Guerrero-Preston R. et al., 2016	***	*	***	7
**4.**	Al-Hebshi et al., 2017	**	*	***	7
**5.**	Banerjee et al., 2017	**	*	**	6
**6.**	Bornigen et al., 2017	**	**	**	6
**7.**	Guerrero-Preston R. et al., 2017	**	**	**	6
**8.**	Lee et al., 2017	**	**	***	7
**9.**	Mok et al., 2017	**	*	**	6
**10.**	Shin et al., 2017	**	**	***	7
**11.**	Zhao et al., 2017	**	**	***	7
**12.**	Hayes R.B. et al., 2018	***	**	***	8
**13.**	Hsiao et al., 2018	**	**	***	7
**14.**	Lim et al., 2018	**	**	***	7
**15.**	Perera et al., 2018	**	**	***	8
**16.**	Vesty et al., 2018	**	**	**	6
**17.**	Chang et al., 2018	**	**	***	7
**18.**	Yang et al., 2018	**	***	***	8
**19.**	Ganly et al., 2019	**	**	**	7
**20.**	Hashimoto et al., 2019	**	**	***	7
**21.**	Panda et al., 2019	**	**	***	7
**22.**	Takahashi et al., 2019	**	**	***	7
**23.**	Zhang et al., 2020	**	**	***	7
**24.**	Zhou et al., 2020	***	**	***	8
**25.**	Sharma et al., 2020	***	**	***	8
**26**	Rai et al., 2020	**	**	***	7
